# Electrochemotherapy in the Management of Keratinocyte Carcinomas: A Systematic Review

**DOI:** 10.3390/cancers17111766

**Published:** 2025-05-24

**Authors:** Yue Ting Nichole Tan, Choon Chiat Oh

**Affiliations:** 1 Duke-NUS Medical School, Singapore 169857, Singapore; tan.nichole@u.duke.nus.edu; 2 Department of Dermatology, Singapore General Hospital, Singapore 169608, Singapore

**Keywords:** basal cell carcinoma, squamous cell carcinoma, keratinocyte carcinoma, skin cancer, electrochemotherapy, electroporation

## Abstract

Background: Keratinocyte carcinomas, including basal cell carcinoma (BCC) and squamous cell carcinoma (SCC), presents a growing concern. Electrochemotherapy (ECT), an emerging treatment modality, combines chemotherapy and electroporation to enhance drug delivery into cells. However, reviews evaluating ECT in keratinocyte carcinomas are lacking. Objectives: This study reviews the efficacy and toxicity of ECT in the treatment and palliation of keratinocyte carcinomas. Methods: A systematic search was conducted across PubMed, Cochrane, Embase, and Scopus databases. Patient, tumor, and treatment characteristics, tumor response, long-term disease outcomes, and toxicity data were extracted. Quality of studies was assessed using validated tools. Primary endpoints included tumor response; secondary endpoints included long-term disease outcomes and toxicity. Results: Twenty-one studies were included. Complete response (CR) rates ranged from 50 to 100% and from 10 to 100% for BCC and SCC, respectively. OS rates ranged from 95 (14 months) to 100 (1 year) % and from 64 (1 year) to 85.1 (8.6 months) % for BCC and SCC, respectively. One-year local disease-free survival (LDFS) rates were 89% and 87% for BCC and SCC, respectively. For BCC, local progression-free survival (LPFS) rates were 96% (1 year), 90% (2 year), and 70% (5 year). For SCC, 1-year LPFS rates were 80% on a per-patient basis and 49% on a per-lesion basis. Conclusions: ECT is effective and tolerable in the treatment and palliation of keratinocyte carcinomas. Future studies should focus on improving reporting quality, optimizing treatment protocols, and investigating long-term outcomes.

## Simple Summary

Electrochemotherapy (ECT) is an emerging treatment modality for skin cancer, yet robust evidence on its role in keratinocyte carcinomas remains limited. Through studies encompassing diverse patient demographics and tumor characteristics, this review demonstrates that ECT is effective and tolerable in the treatment and palliation of keratinocyte carcinomas. This review also provides guidance for future research, emphasizing the need to enhance reporting quality, optimize treatment protocols, and investigate long-term outcomes.

## 1. Introduction

Keratinocyte carcinomas, including basal cell carcinoma (BCC) and squamous cell carcinoma (SCC), are among the most common malignancies in White populations [1]. Their incidence is on the rise, driven by prolonged ultraviolet (UV) exposure, global warming, and an aging population [2].

Surgical resection remains the standard treatment for primary skin malignancies [3], but its feasibility is dependent on tumor characteristics and patient co-morbidities. Additionally, resistance to standard treatments is growing, with locally advanced and metastatic BCC and SCC showing increasing resistance to traditional therapies [4,5,6,7]. BCC has also developed resistance to newer therapies such as hedgehog inhibitors [8]. Moreover, toxicities associated with systemic chemotherapy and immunotherapy can negatively impact patients’ quality of life (QoL) [9].

Given these challenges, there is an increasing need for alternative treatment modalities that can provide effective tumor control while minimizing systemic side effects. Electrochemotherapy (ECT) combines chemotherapy and electroporation, utilizing electric pulses to increase the permeability of cancer cell membranes to chemotherapeutic agents such as bleomycin and cisplatin [10,11,12,13]. First conceptualized in the 1980s, ECT is a relatively new cancer treatment modality [14]. Originally indicated for inoperable skin cancer, it is now also used in the treatment and palliation of skin metastases [12,15]. ECT also confers local cancer control with minimal damage to surrounding tissues [16,17]. However, treatment protocols have varied across studies, particularly in terms of the chemotherapeutic agent used, electrode type, and electric pulse parameters. These variations may influence treatment efficacy, contributing to differences in clinical outcomes reported across studies. To standardize clinical practice, the European Standard Operating Procedures in Electrochemotherapy (ESOPE) guidelines were developed to ensure that the use of ECT in the management of skin cancer is consistent and optimized [18].

Recent reviews and meta-analyses on ECT in metastatic melanoma have reported overall response (OR) rates of 77.6–80.6% and 1-year overall survival (OS) rates of 67–89% [19,20]. However, reviews focusing specifically on the effects of ECT in keratinocyte carcinomas are sparse. A previous review on ECT in BCC found outcomes comparable to those achieved with conventional surgery, but studies included were few and with heterogenous methods of data reporting [21]. No reviews have also examined the role of ECT in SCC.

Therefore, this study aims to systematically review the current literature on the efficacy and toxicity of ECT in the treatment and palliation of keratinocyte carcinomas. Variables including patient and tumor characteristics, treatment parameters, tumor response, long-term disease outcomes, and treatment-related toxicities will be assessed to identify patterns influencing treatment outcomes and to highlight areas of variability across studies in order to inform future research and optimization of ECT protocols in the management of BCC and SCC.

## 2. Materials and Methods

### 2.1. Search Strategy and Study Selection

A systematic search of the PubMed, Cochrane, Embase, and Scopus databases from the earliest date to 24 March 2024 was performed using search terms (‘electrochemotherapy’ AND (‘skin’ AND ‘cancer’)). Retrospective and prospective studies and clinical trials published with full text in English between 1 January 2014 to 31 December 2023 on ECT in the management of primary and metastatic BCC and SCC were included. Systematic or narrative reviews, meta-analyses, case reports, letter-commentaries-editorials, meeting abstracts, and guidelines-recommendations were excluded. Studies with a sample size of <10, in vitro and veterinary studies, as well as studies which lacked reporting on tumor response or long-term disease outcomes were also excluded. Two reviewers (NT, CC) independently reviewed records, removed duplicates, and selected articles at the title/abstract level. Discrepancies were resolved by discussion. Protocol for this review was registered with PROSPERO (registration number CRD42024567182).

### 2.2. Data Extraction

The data were extracted from studies on the use of ECT among patients with BCC and SCC. In studies that included patients with other non-keratinocyte skin cancers, data specific to BCC and SCC were extracted, wherever possible. The data included patient and tumor characteristics (age, site, size, and number of tumors, type and stage of cancer), ECT characteristics (drug, dose, and route of administration, electroporator, electrode, number of cycles, type of anesthesia), and the presence of previous or concurrent therapies. The data on tumor response after the first ECT cycle in terms of complete response (CR), partial response (PR), stable disease (SD), and progressive disease (PD) rates, according to the Response Evaluation Criteria in Solid Tumors (RECIST), were extracted. Duration of follow-up and long-term disease outcomes, including OS, local progression-free survival (LPFS) and local disease-free survival (LDFS) rates, and treatment toxicity were extracted.

### 2.3. Quality Assessment

Quality of studies was assessed using the Version 1 of the Risk of Bias in Nonrandomized Studies of Interventions (ROBINS-I) assessment tool [22] for observational studies, and the Version 2 of the Cochrane risk-of-bias tool for randomized trials (RoB 2) assessment tool for randomized controlled trials (RCTs) [23], as recommended by the Cochrane Handbook [24]. The ROBINS-I tool evaluates bias across seven domains, including the risk of bias due to confounding, deviations from intended interventions, missing data, and the risk of bias in the selection of participants, classification of interventions, measurement of outcomes, and selection of the reported result. Studies were then categorized as having low, moderate, serious, or critical risk of bias. The RoB 2 tool evaluates bias across five domains, including the risk of bias due to deviations from the intended intervention and due to missing outcome data, the risk of bias in the measurement of the outcome and the selection of reported results, and the risk of bias arising from the randomization process. Studies were then categorized as having a low risk of bias, some concerns, or a high risk of bias.

### 2.4. Statistical Analysis

The primary endpoints were tumor response to ECT in terms of CR, PR, SD, and PD rates. The secondary endpoints were long-term disease outcomes in terms of OS, LPFS, and LDFS rates, and treatment toxicity.

## 3. Results

### 3.1. Included Studies

**Figure 1:**
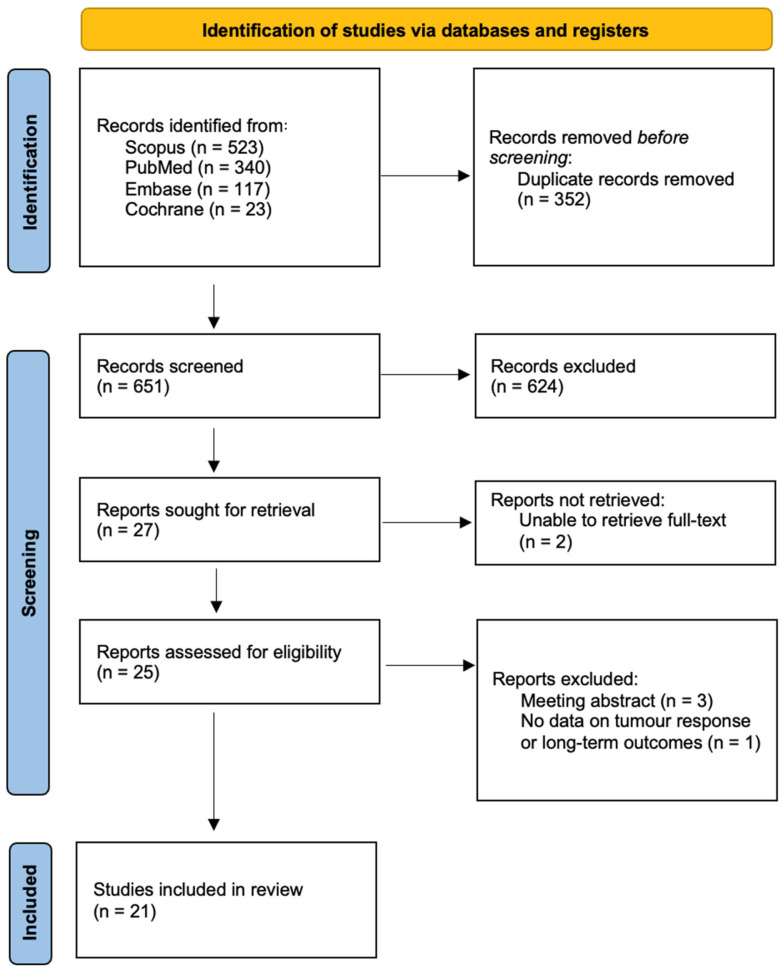
PRISMA flow chart diagram of the search strategy.

The search results are shown in the Preferred Reporting Items for Systematic Reviews and Meta-analyses (PRISMA) flow-chart (Figure 1). From an initial search of 1003 records and after removing duplicates, 651 were screened at the title/abstract level. In total, 624 were excluded for not meeting the inclusion criteria; two lacked full text; 3 were meeting abstracts; and 1 lacked data on tumor response to ECT and long-term disease outcomes. A total of 21 studies were included: 1 RCT [25], 3 single-arm trials [26,27,28] and 10 prospective [16,29,30,31,32,33,34,35,36,37] and 7 retrospective [38,39,40,41,42,43,44] cohort studies.

### 3.2. Risk of Bias of Included Studies

Three observational studies had a serious risk of bias: two from confounding [34,44] and one from missing data [27]. Apart from one study, which had low risk [32], the rest of the observational studies had a moderate risk of bias. The RCT had some risk of bias arising from deviations from the intended deviation, missing outcomes, and from outcome measurement [25].

### 3.3. Patient and Tumor Characteristics

Patient and tumor characteristics for BCC and SCC are presented in Table 1 and Table 2, respectively.

A total of 1890 patients, aged 11–104, were included. Four studies included only patients with BCC [25,29,31,41], two included only SCC [37,44], and two included only BCC and SCC [32,33]. The remaining included other skin cancers. Four treated patients with palliative intent [26,28,38,41]; the rest with curative intent.

Apart from five studies which did not report on the number of treated tumors [35,38,39,40,43], 5629 tumors in the head-and-neck region, trunk, and extremities, were treated and ranged from 1 to 60 per patient. Tumor diameters ranged from 2 to 500 mm.

### 3.4. Treatment Characteristics

Treatment characteristics for BCC and SCC are presented in Table 3 and Table 4, respectively. While most patients included in the studies had previously undergone treatments such as surgery, chemotherapy, radiotherapy, and immunotherapy, ECT was used as the primary treatment modality during the study period in most patients where it was administered as monotherapy, except for in two studies where it was combined with other therapies such as surgery [30], immunotherapy, chemotherapy, and photodynamic therapy [26]. One study used the ePORE electroporator (Mirai Medical, Galway) [27] and another used the Sennex electroporator (BIONMED Technologies GmbH, Saarbrücken, Germany) [43], while the rest used the Cliniporator (IGEA GmbH, Frankfurt am Main, Germany). All studies delivered ECT in accordance with the ESOPE guidelines [18], except for one, which used high-frequency electroporation instead of the traditional low-frequency electroporation [27].

Except one study which also used cisplatin [30], all exclusively used bleomycin. Bleomycin was typically administered intravenously at 15,000 IU/m^2^ (range: 7500–30,000 IU/m^2^). Two compared reduced- and standard-dose bleomycin (10,000 vs. 15,000 IU/m^2^) [32,33]. When administered intratumorally, the typical dose was 1000 IU (range: 250–5000 IU). Electrodes were mainly arranged in hexagonal and linear arrays. Most patients received one ECT cycle (range: 1–6).

### 3.5. Outcomes

#### 3.5.1. Tumor Response

Tumor response, long-term disease outcomes, and toxicity data for BCC and SCC are presented in Table 5 and Table 6, respectively. All studies used the RECIST scale to evaluate tumor response. Response was typically evaluated at 30–90 days post-ECT. CR rates ranged from 50 to 100% in BCC and 10–100% in SCC. Most reported a PD rate of 0%, with the highest rate at 9.5% in a study involving SCC patients with tumors greater than 3 cm in diameter [16].

Follow-up duration ranged from 165 days to up to 5 years. OS rates ranged from 95% (14 months) to 100% (1 year) among BCC patients [16,37], and from 64% (1 year) to 85.1% (8.6 months) among SCC patients [16,37]. One-year LDFS rates were reported at 89% for BCC, and 87% for SCC [16]. For BCC, LPFS rates ranged from 96% (1 year) to 90% (2 year) and to 70% (5 year) [31,41]. For SCC, 1-year LPFS rates were reported at 80% on a per-patient basis and 49% on a per-lesion basis [37].

#### 3.5.2. Internal Comparisons

An RCT compared ECT to surgical excision and found similar CR rates in patients with primary BCC [25]. Another study also compared reduced- and standard-dose bleomycin in BCC and SCC patients and found similar CR rates [33]. Another compared outcomes between patients aged <90 years and ≥90 years but did not report outcomes specifically pertaining to BCC and SCC [39].

#### 3.5.3. Toxicity

Eleven studies evaluated adverse events with the Common Terminology Criteria for Adverse Events (CTCAE) [16,25,30,31,33,35,37,39,40,41,44] and five studies reported on the most frequent adverse events [26,29,38,42,43], with pain hyperpigmentation, and erythema being the most common toxicities.

## 4. Discussion

These studies highlight the broad applicability of ECT in treating keratinocyte carcinomas, featuring diverse patient and tumor characteristics. Although the recent registry-based studies in this review demonstrate its real-world efficacy and tolerability [31,35,37], there remains a paucity of RCTs comparing ECT to other established treatments.

Currently, ECT is recognized for treating various tumor histotypes and palliating skin metastases. However, only a minority of patients in the studies reviewed were treated with palliative intent, limiting definitive conclusions on its palliative role.

All studies reviewed treated head-and-neck tumors, which presents unique challenges due to the anatomical complexity of the region and the risk of cutaneous ECT-related side effects. Since BCC and SCC are commonly located in the head-and-neck region [45], these studies improve our understanding of using ECT to treat these tumors.

Studies varied in drug and electrode usage, anesthesia, number of ECT cycles, and the presence of prior and concurrent treatments. Most used intratumoral or intravenous bleomycin, with one using intratumoral cisplatin. According to the ESOPE guidelines, intravenous injection is only recommended for bleomycin, possibly explaining its more widespread use [18]. However, cisplatin remains an alternative for patients where the use of systemic bleomycin may be unfavorable, such as those with pulmonary or renal disease due to its toxicities [46].

Two studies investigated reduced bleomycin doses [32,33] and one found no significant difference in tumor control among elderly BCC and SCC patients [33]. These findings are supported by a prior bleomycin pharmacokinetics-based study which found equivalence between standard and reduced doses in elderly patients due to reduced renal clearance with age [47].

Studies mostly used needle electrodes of varying arrays, primarily in linear and hexagonal configurations. According to the updated ESOPE guidelines, linear array electrodes are recommend for smaller tumors, particularly in the head-and-neck region, as their relatively lower applied voltage results in minimal or no hyperpigmentation [48]. In contrast, hexagonal array electrodes are preferred for larger tumors, given their ability to cover a wider treatment area [48].

Outcomes varied based on tumor size. One study reported an OR rate of 66.7% among BCC patients with larger tumors (>3 cm) compared to 93.5% among those with smaller tumors (≤3 cm) [16]. A similar trend was observed in SCC patients (28.6% vs. 76.9%) [16]. On the other hand, studies treating smaller tumors with median diameters of 1–2.5 cm reported OR rates of 100% among SCC patients [33] and 100% and 96% among BCC patients receiving reduced- and standard-dose bleomycin, respectively [33]. Another study treated smaller tumors with mean volumes of 3206 mm^3^ in BCC and 2055 mm^3^ in SCC, and reported OR rates of 85% and 100%, respectively [27]. These findings are consistent with previous research, which reported lower response rates in larger tumors [36,49], possibly due to reduced drug penetration and challenging electrode placement. The updated ESOPE guidelines therefore recommend the use of hexagonal electrodes for larger tumors due to the relatively larger area covered [48]. In addition, intravenous bleomycin has also shown superior outcomes compared to intratumoral administration for larger tumors [50].

Prior treatments may also influence outcomes of ECT. Higher OR rates were reported in treatment-naïve BCC and SCC patients in two studies [16,31], noting previous chemoradiotherapy affected ECT outcomes more than surgery. Another study also reported lower CR rates in BCC patients with prior irradiation [25], consistent with what have previously been reported among melanoma patients [20].

Few studies included patients who received concurrent treatment. One studied patients who underwent concurrent surgery [30], and another studied patients who received concurrent treatments including immunotherapy, photodynamic therapy, and chemotherapy [26]. However, the influence of concurrent therapies on outcomes of ECT remains unclear as subgroup analyses comparing outcomes with those receiving only ECT was not performed in those studies.

The longest follow-up duration was reported in a study among BCC patients, with a 5-year LPFS rate of 70% [41]. Better outcomes were also associated with small tumor size, early cancer stage and localized disease [41]. Likewise, a study among SCC patients reported that 1-year LPFS was significantly higher in patients with primary lesions compared to those with locally advanced disease (80% vs. 49%) [37]. A study involving patients treated with palliative intent reported lower OS rates, with a 1-year OS of 46.5% [26].

Most ECT-related side effects were pain, erythema, and hyperpigmentation but reporting was variable, complicating comparisons across studies. Notably, increased local toxicities including necrosis and ulceration were observed in studies where ECT was combined with chemotherapy or immunotherapy, or when hexagonal electrodes were used [51,52,53]. Previous research has also reported an association between concurrent radiation therapy with fibrosis and vascular damage [54]. Patients’ age may also influence the occurrence of side effects. One study compared outcomes between patients aged <90 years and ≥90 years and noted prolonged wound healing in those aged ≥90 years, suggesting that ECT might therefore benefit older patients who are more prone to side effects from traditional treatments [39].

This review has some limitations. Most studies included had a moderate to serious risk of bias, and studies were heterogenous and incomplete in their reporting of patient and treatment characteristics and outcomes. In addition, some studies reported outcomes for other skin cancer types, including non-keratinocyte carcinomas, without distinguishing results for BCC and SCC. In this regard, a previous review has outlined recommendations for improving reporting quality in ECT studies, which future studies may adopt [55]. Additionally, long-term survival data were not reported by most studies. As ECT is a relatively new treatment modality, long-term data are crucial to assess its outcomes over longer durations.

Future studies should include more homogenous patient groups to identify subgroups that benefit most from ECT. Currently, most studies included patients with various tumor histotypes. This limits the ability to perform subgroup analyses and hinders clear outcomes related to specific cancer types. More comparative studies are also required to understand how patient and treatment factors influence outcome in order to refine protocols to maximize efficacy while minimizing toxicities.

## 5. Conclusions

This review highlights the broad applicability, effectiveness, and tolerability of ECT in the treatment and palliation of keratinocyte carcinomas. It also underscores the need for more RCTs to compare ECT with other established treatment modalities. Future studies should focus on improving reporting quality, optimizing treatment protocols, and investigating long-term outcomes.

## Figures and Tables

**Table 1:** Patient and tumor characteristics for basal cell carcinoma (BCC).

Study	Design	Disease Stage	Treatment Intent	Number of Participants Treated	Age, Median (Range), Years	Number of Tumors Treated	Tumors per Patient, Median (Range), Number	Site of Tumors	Dimension of Tumors, Median (Range)
Kis et al. 2019 [29]	Prospective	NR	Curative	12	61.6 ^a^ (11–86)	15	1 (1–2)	H-N, trunk, extremities	12 mm (2–43)
Clover et al. 2020 [25]	Randomized controlled trial	NR	Curative	ECT: 50 Surgery: 42	ECT: 66.8 (24–92) ^a, b^ Surgery: 63.8 (37–91) ^a, b^	ECT: 69 Surgery: 48	1 (81.5%), 2 (14.1%), 3 (3.3%), 7 (1.1%)	NR	ECT: 17 mm^2^ (2.4–105) Surgery: 14 mm^2^ (2.5–5)
Campana et al., 2016 [30]	Prospective	IV (38.3%) ^c^	Curative	24	71 (24–100) ^c^	1304 ^c^	3 (1–5) ^c^	H-N, trunk, extremities	10 mm (6–20) ^c^
Bertino et al. 2022 [31]	Prospective	NR	Curative	330	72 (23–98)	623	1 (1–7)	H-N, trunk, extremities	13 mm (5–350)
Riva et al. 2021 [38]	Retrospective	NR	Palliative	4	78 ^c^	NR	NR	H-N	<30 mm: 27% >30 mm: 63%
Sersa et al. 2021 [39]	Retrospective	NR	Curative	≥90 years: 11 <90 years: 22	≥90 years: 92 (90–104) ^c^ <90 years: 77 (23–89) ^c^	NR	1 (1–7) ^c^	H-N, trunk, extremities	≥90 years: 15 mm (5–450) ^c^ <90 years: 15 mm (5–500) ^c^
Jamsek et al. 2020 [32]	Prospective	NR	Curative	Reduced dose: 10 Standard dose: 14	Reduced dose: 81.5 (67–92) Standard dose: 79.5 (65–89)	Reduced dose: 17 Standard dose: 25	Reduced dose: 1.5 (1–4) Standard dose: 1 (1–5)	H-N	Reduced dose: 15 mm (7–80) Standard dose: 11 mm (4–50)
Bonadies et al. 2019 [40]	Retrospective	NS	Curative	3	80 (30–102) ^c^	NR	1 (37%), ≥2 (63%) ^c^	H-N, trunk, extremities	NR
Groselj et al. 2018 [33]	Prospective	NR	Curative	Reduced dose: 10 Standard dose: 13	Reduced dose: 81.5 (67–92) Standard dose: 79.5 (65–89)	Reduced dose: 16 Standard dose: 25	Reduced dose: 1.5 (1–4) Standard dose: 1 (1–5)	H-N	Reduced dose: 15 mm (7–80) Standard dose: 11 mm (4–50)
Bertino et al., 2016 [16]	Prospective	I–II (83.8%), III–IV (16.2%) ^c^	Curative	34	77 (39–96) ^c^	34	1 (1)	H-N	≤30 mm: 91.2% >30 mm: 8.8%
Tomassini et al., 2016 [34]	Prospective	NR	Curative	4	85 (40–95)	19 ^c^	NR	H-N	110 mm (30–180) ^d^
Claussen et al. 2022 [35]	Prospective	NR	Curative	193	72 ^a, c^	1784 ^c^	NR	H-N, trunk, extremities	Non-ulcerated lesions: 15 mm (5–450) ^a^ Ulcerated lesions: 30 mm (5–500) ^a^
Lyons et al. 2023 [27]	Single-arm trial	NR	Curative	25 ^c^	73.6 ^a, c^	30	NR	NR	3206 mm^3 a^
Clover et al. 2020 [36]	Prospective	NR	Curative	298	75 (20–104) ^c^	567	NR	H-N, trunk, extremities	23 mm (5–500) ^c^
Campana et al. 2017 [41]	Retrospective	I–III (96%), IV (4%)	Palliative	84	69 (24–89)	185	2 (1–3)	H-N, trunk, extremities	20 mm (5–267)
Rotunno et al. 2018 [42]	Retrospective	NR	Curative	10	78 (43–97) ^c^	147 ^c^	2 (1–7) ^c^	H-N, trunk, extremities	10 mm (5–190) ^c^
Solari et al. 2014 [28]	Single-arm trial	NR	Palliative	2	72 (47–91) ^c^	NR	20 (1–60) ^c, e^	H-N, trunk, extremities	< 20 mm: 43.6% ^c^ ≥ 20 mm: 56.4% ^c^

ECT: electrochemotherapy; H-N: head-and-neck; NR: not reported. ^a^ Data presented as mean; ^b^ Only data on enrolled patients available (ECT = 52; Surgery = 48). ^c^ The data specific to BCC are not available and encompasses a broader category of skin cancers as reported in the individual studies. ^d^ The sum of the longest diameters. ^e^ A total of 25 (64.1%) patients had more than 10 tumors and 14 (35.9%) patients had 10 or less tumors.

**Table 2:** Patient and tumor characteristics for squamous cell carcinoma (SCC).

Study	Design	Disease Stage	Mucosal/Cutaneous	Treatment Intent	Number of Participants Treated	Age, Median (Range), Years	Number of Tumors Treated	Tumors per Patient, Median (Range), Number	Site of Tumors	Dimension of Tumors, Median (Range)
Campana et al., 2016 [30]	Prospective	IV (38.3%) ^a^	Cutaneous	Curative	41	71 (24–100) ^a^	1304 ^a^	3 (1–5) ^a^	H-N, trunk, extremities	10 mm (6–20) ^a^
Bertino et al. 2022 [37]	Prospective	NS	Cutaneous	Curative	162	80 (41–104)	342	1 (1–7)	H-N, trunk, extremities	21 mm (5–250)
Riva et al. 2021 [38]	Retrospective	NR	Cutaneous and mucosal	Palliative	18	78 ^a^	NR	NR	H-N	<30 mm: 27% >30 mm: 63%
Sersa et al. 2021 [39]	Retrospective	NR	Cutaneous	Curative	≥90 years: 16 <90 years: 32	≥90 years: 92 (90–104) ^a^ <90 years: 77 (23–89) ^a^	NR	1 (1–7) ^a^	H-N, trunk, extremities	≥90 years: 15 mm (5–450) ^a^ <90 years: 15 mm (5–500) ^a^
Jamsek et al. 2020 [32]	Prospective	NR	Cutaneous	Curative	Reduced dose: 3 Standard dose: 3	Reduced dose: 82 (76–83) Standard dose: 85 (67–89)	Reduced dose: 7 Standard dose: 3	Reduced dose: 3 (1–3) Standard dose: 1 (1–2)	H-N	Reduced dose: 10 mm (6–35) Standard dose: 25 mm (22–45)
Bonadies et al. 2019 [40]	Retrospective	NS	Cutaneous	Curative	12	80 (30–102) ^a^	NR	1 (37%), ≥2 (63%) ^a^	H-N, trunk, extremities	NR
Pichi et al. 2018 [26]	Single-arm trial	IV (16.7%)	Cutaneous and mucosal	Palliative	20	72.5 (52–92)	NR	NR	H-N	NR
Groselj et al. 2018 [33]	Prospective	NR	NS	Curative	Reduced dose: 3 Standard dose: 3	Reduced dose: 82 (76–83) Standard dose: 85 (67–89)	Reduced dose: 8 Standard dose: 3	Reduced dose: 3 (2–3) Standard dose: 1 (1–2)	H-N	Reduced dose: 20 mm (6–35) Standard dose: 25 mm (22–45)
Bertino et al., 2016 [16]	Prospective	I–II (83.8%), III–IV (16.2%) ^a^	NS	Curative	50	77 (39–96) ^a^	50	1 (1)	H-N	≤30 mm: 52% >30 mm: 48%
Tomassini et al., 2016 [34]	Prospective	NR	Cutaneous	Curative	2	78.5 (75–82)	19 ^a^	NR	H-N	110 mm (30–180) ^b^
Kreuter et al. 2015 [43]	Retrospective	III–IV (100%)	Cutaneous	Curative	5	73.1 ^a^	NR	NR	H-N, trunk, extremities	NR
Claussen et al. 2022 [35]	Prospective	NR	Cutaneous	Curative	129	72 ^a, c^	1784 ^a^	NR	H-N, trunk, extremities	Non-ulcerated lesions: 15 mm (5–450) ^a^ Ulcerated lesions: 30 mm (5–500) ^a^
Lyons et al. 2023 [27]	Single-arm trial	NR	NR	Curative	25 ^a^	73.6 ^a, c^	2	NR	NR	2055 mm^3 c^
Clover et al. 2020 [36]	Prospective	NR	NR	Curative	156	75 (20–104) ^a^	284	NR	H-N, trunk, extremities	23 mm (5–500) ^a^
Rotunno et al. 2018 [42]	Retrospective	NR	NR	Curative	13	78 (43–97) ^a^	147 ^a^	2 (1–7) ^a^	H-N, trunk, extremities	10 mm (5–190) ^a^
Di Monta et al. 2017 [44]	Retrospective	III (100%)	Cutaneous	Curative	22	72 (51–88)	22	1 (1)	H-N, trunk, extremities	NR
Solari et al. 2014 [28]	Single-arm trial	NR	NR	Palliative	5	72 (47–91) ^a^	NR	20 (1–60) ^a, d^	H-N, trunk, extremities	<20 mm: 43.6% ^a^ ≥20 mm: 56.4% ^a^

H-N: head-and-neck; NR: not reported. ^a^ The data specific to SCC are not available and encompasses a broader category of skin cancers as reported in the individual studies. ^b^ The sum of the longest diameters. ^c^ The data are presented as mean. ^d^ A total of 25 (64.1%) patients had more than 10 tumors and 14 (35.9%) patients had 10 or less tumors.

**Table 3:** Treatment characteristics for basal cell carcinoma (BCC).

Study	Drug	Route	Dose (Range)	Electrode	Anesthesia	Number of Cycles	Previous Therapies	Concurrent Therapies
Kis et al. 2019 [29]	BLM	IV (75%), IT (25%)	NR	Needle (hexagonal), row	GA	1–5	Surgery, IMT (75%)	None
Clover et al. 2020 [25]	BLM	IT	1653 IU (500–5000)	Needle (hexagonal), parallel	LA, GA	1–2	NR	None
Campana et al., 2016 [30] ^a^	BLM, CDDP	IV BLM (93.4%), IT BLM (6.1%), IV CDDP (0.5%)	NR	Needle (hexagonal, linear), plate, multiple	LA, GA	1 (76.3%), 2 (19.1%), 3 (3.5%), 3 (0.8%), 6 (0.3%)	Surgery, CHT, RT, IMT (81.4%)	Surgery (6.1%)
Bertino et al. 2022 [31]	BLM	IV (56%), IT (44%)	IV: 15,000–30,000 IU/m^2^ IT: 1000 IU	Needle (hexagonal), row, plate	LA, GA	1 (84%), 2 (16%)	Surgery, RT, CYT, PDT, ECT, TT (39%)	None
Riva et al., 2021 [38] ^a^	BLM	IV	15,000 IU/m^2^	Needle (linear), finger	NR	NR	RT (44.4%)	None
Sersa et al. 2021 [39] ^a^	BLM	≥90 years: IV (66%), IT (34%) <90 years: IV (73%), IT (27%)	NR	Needle (hexagonal, linear), plate, multiple	LA, GA	≥90 years: 1 (90%), 2 (10%) <90 years: 1 (89%), 2 (11%)	≥90 years: Surgery, RT, CYT, PDT (51%) <90 years: Surgery, RT, CYT, PDT (56%)	None
Jamsek et al. 2020 [32] ^a^	BLM	IV	Reduced dose: 10,000 IU/m^2^ Standard dose: 15,000 IU/m^2^	Plate, needle (linear, hexagonal)	LA, GA	NR	NR	NR
Bonadies et al. 2019 [40] ^a^	BLM	IV	15,000 IU/m^2^	Plate, finger, needle (linear, hexagonal)	NR	1 (48%), 2 (37%), 3 (12%), 6 (3%)	Surgery, CHT, PDT (67%) None (33%)	None
Groselj et al. 2018 [33] ^a^	BLM	IV	Reduced dose: 10,000 IU/m^2^ Standard dose: 15,000 IU/m^2^	Plate, needle (linear, hexagonal)	LA, GA	NR	Surgery, RT (25%)	None
Bertino et al., 2016 [16] ^a^	BLM	IV (92%), IT (8%)	NR	Plate, needle (hexagonal), row, combination	LA, GA	1 (82%), 2 (18%)	Surgery (31%), CHT/RT (9%), surgery with CHT/RT (31%), unknown (2%)	None
Tomassini et al., 2016 [34] ^a^	BLM	IV	15,000 IU/m^2^	Finger	LA	1 (53.8%), 2 (46.2%)	NR	None
Claussen et al. 2022 [35] ^a^	BLM	IV, IT	IV: 15,000 IU/m^2^ IT: 1000 IU	Needle (linear, hexagonal), plate	NR	NR	NR	None
Lyons et al. 2023 [27] ^a^	BLM	IV, IT	IV: 15,000 IU/m^2^ IT: 1000 IU	NR	LA, GA, spinal anesthesia	NR	NR	None
Clover et al. 2020 [36] ^a^	BLM	IV (75%), IT (25%)	IV: 15,000 IU/m^2^ IT: 1000 IU	Plate, needle (hexagonal), row, combination	LA, GA, regional anesthesia	NR	NR	None
Campana et al. 2017 [41]	BLM	IV, IT	IV: 15,000 IU/m^2^ IT: 250–1000 IU	Needle (linear, hexagonal), finger	LA, GA, sedation, general	1 (71.4%), 2 (27.4%), 4 (1.2%)	Surgery (46%), RT (24%), IMT (7%), PDT (2.4%), CYT (6.0%), TT (1.2%)	None
Rotunno et al. 2018 [42] ^a^	BLM	IV	IV: 7500, 10,000, 13,500 IU/m^2^	Needle (linear, hexagonal), plate, multiple	LA, GA, regional	1 (74%), 2 (19%), 3 (7%)	RT (6.8%)	None
Solari et al. 2014 [28] ^a^	BLM	IV	15,000 IU/m^2^	Needle (hexagonal)	GA	1 (56.4%), 2 (30.8%), 3 (10.3%), 4 (4.5%)	NR	None

BLM: bleomycin; CDDP: cisplatin; CHT: chemotherapy; CYT: cryotherapy; GA: general anesthesia; IV: intravenous; IT: intratumoral; IMT: immunotherapy; LA: local anesthesia; NR: not reported; PDT: photodynamic therapy; RT: radiotherapy; TT: topical therapies. ^a^ The data specific to BCC are not available and encompasses a broader category of skin cancers as reported in the individual studies.

**Table 4:** Treatment characteristics for squamous cell carcinoma (SCC).

Study	Drug	Route	Dose (Range)	Electrode	Anesthesia	Number of Cycles	Previous Therapies	Concurrent Therapies
Campana et al., 2016 [30] ^a^	BLM, CDDP	IV BLM (93.4%), IT BLM (6.1%), IV CDDP (0.5%)	NR	Needle (hexagonal, linear), plate, multiple	LA, GA	1 (76.3%), 2 (19.1%), 3 (3.5%), 3 (0.8%), 6 (0.3%)	Surgery, CHT, RT, IMT (81.4%)	Surgery (6.1%)
Bertino et al. 2022 [37]	BLM	IV (83%), IT (17)	IV: 15,000 IU/m^2^ IT: 1000 IU	Needle (hexagonal, linear), plate, multiple	LA, GA	1 (90.1%), 2 (9.3%), 3 (0.6%)	Surgery, RT, CHT, CYT, PDT, IMT, ECT (70%)	None
Riva et al., 2021 [38] ^a^	BLM	IV	15,000 IU/m^2^	Needle (linear), finger	NR	NR	RT (44.4%)	None
Sersa et al. 2021 [39] ^a^	BLM	≥90 years: IV (66%), IT (34%) <90 years: IV (73%), IT (27%)	NR	Needle (hexagonal, linear), plate, multiple	LA, GA	≥90 years: 1 (90%), 2 (10%) <90 years: 1 (89%), 2 (11%)	≥90 years: Surgery, RT, CYT, PDT (51%) <90 years: Surgery, RT, CYT, PDT (56%)	None
Jamsek et al. 2020 [32] ^a^	BLM	IV	Reduced dose: 10,000 IU/m^2^ Standard dose: 15,000 IU/m^2^	Plate, needle (linear, hexagonal)	LA, GA	NR	NR	NR
Bonadies et al. 2019 [40] ^a^	BLM	IV	15,000 IU/m^2^	Plate, finger, needle (linear, hexagonal)	NR	1 (48%), 2 (37%), 3 (12%), 6 (3%)	Surgery, CHT, PDT (67%)	None
Pichi et al. 2018 [26]	BLM	IV	15,000 IU/m^2^	Finger, needle (hexagonal)	LA	1 (60%), 2 (25%), 3 (10%), 4 (5%)	NR	IMT with cetuximab, PDT, CHT with MTX (20%)
Groselj et al. 2018 [33] ^a^	BLM	IV	Reduced dose: 10,000 IU/m^2^ Standard dose: 15,000 IU/m^2^	Plate, needle (linear, hexagonal)	LA, GA	NR	Surgery, RT (25%)	None
Bertino et al., 2016 [16] ^a^	BLM	IV (92%), IT (8%)	NR	Plate, needle (hexagonal), row, combination	LA, GA	1 (82%), 2 (18%)	Surgery (31%), CHT/RT (9%), surgery with CHT/RT (31%), unknown (2%)	None
Tomassini et al., 2016 [34] ^a^	BLM	IV	15,000 IU/m^2^	Finger	LA	1 (53.8%), 2 (46.2%)	NR	None
Kreuter et al. 2015 [43] ^a^	BLM	IV	NR	Needle (linear, hexagonal), plate	NR	2.1 ^b^	Surgery, RT, CHT ^c^	None
Claussen et al. 2022 [35] ^a^	BLM	IV, IT	IV: 15,000 IU/m^2^ IT: 1000 IU	Needle (linear, hexagonal), plate	NR	NR	NR	None
Lyons et al. 2023 [27] ^a^	BLM	IV, IT	IV: 15,000 IU/m^2^ IT: 1000 IU	NR	LA, GA, spinal anesthesia	NR	NR	None
Clover et al. 2020 [36] ^a^	BLM	IV (75%), IT (25%)	IV: 15,000 IU/m^2^ IT: 1000 IU	Plate, needle (hexagonal), row, combination	LA, GA, regional anesthesia	NR	NR	None
Rotunno et al. 2018 [42] ^a^	BLM	IV	IV: 7500, 10,000, 13,500 IU/m^2^	Needle (linear, hexagonal), plate, multiple	LA, GA, regional	1 (74%), 2 (19%), 3 (7%)	RT (6.8%)	None
Di Monta et al. 2017 [44]	BLM	IV	15,000 IU/m^2^	Needle (linear)	GA	1 (68.2%), 2 (27.3%), 3 (4.5%)	NR	None
Solari et al. 2014 [28] ^a^	BLM	IV	15,000 IU/m^2^	Needle (hexagonal)	GA	1 (56.4%), 2 (30.8%), 3 (10.3%), 4 (4.5%)	NR	None

BLM: bleomycin; CDDP: cisplatin; CHT: chemotherapy; CYT: cryotherapy; GA: general anesthesia; IV: intravenous; IT: intratumoral; IMT: immunotherapy; LA: local anesthesia; NR: not reported; PDT: photodynamic therapy; RT: radiotherapy; TT: topical therapies. ^a^ The data specific to SCC are not available and encompasses a broader category of skin cancers as reported in the individual studies. ^b^ The data are presented as mean. ^c^ The proportion of patients is not reported.

**Table 5:** Tumor response and toxicity for basal cell carcinoma (BCC).

Study	Response Scale	Response Evaluation	Time of Response Evaluation	Follow-Up Duration, Median (Range)	CR (%)	PR (%)	SD (%)	PD (%)	Toxicity Scale	Toxicity	OS	Local Tumour Control
Kis et al. 2019 [29]	RECIST	Patient	NR	19 months (15–56)	58.3% ^a^	NR	NR	NR	NR	Hyperemia and edema (80%), pain (50%)	NR	NR
Clover et al. 2020 [25]	RECIST	Patient/Lesion	60 days	Up till 5 years	ECT: 88.9%/88.4% ^b^ Surgery: 95.1%/95.8% ^c^	NR	NR	NR	CTCAE	ECT: Infection, ulceration, erythema, pain Surgery: Infection, erythema, swelling	NR	NR
Campana et al. 2016 [30]	RECIST	Patient	60 days	13.9 months (0.4–63.2)	66.7%	NR	NR	NR	CTCAE	Grade 0–1 (100%), 2–4 (0%)	NS	NS
Bertino et al. 2022 [31]	RECIST	Patient/Lesion	60 days	17 months (2–103)	81.5% ^e^/84.3% ^f^	15.5% ^e^/13.1% ^f^	3.0% ^e^/2.6% ^f^	0% ^e^/0% ^f^	CTCAE	Hyperpigmentation, ulceration	14-month: 95% ^g^	1-year LPFS: 96% 2-year LPFS: 90%
Riva et al., 2021 [38] ^d^	RECIST	Patient	1 month	Up till 6 months	NS	NS	NS	NS	NR	Edema	NR	NR
Sersa et al. 2021 [39] ^d^	RECIST	Patient	38 and 80 days	≥90 years: 8 months (2–37) <90 years: 9 months (2–46)	NS	NS	NS	NS	CTCAE	Ulceration, odor, infection, hyperpigmentation	NS	NS
Jamsek et al. 2020 [32] ^d^	RECIST	Patient	2 months	Reduced dose: 28 months Standard dose: 40 months	Reduced dose: 100% Standard dose: 96%	NR	NR	NR	NR	NR	NR	NR
Bonadies et al. 2019 [40]	RECIST	Patient	2 months	NR	100%	0%	NR	NR	CTCAE	Necrosis, edema ^d^	NR	NS
Groselj et al. 2018 [33]	RECIST	Lesion	2 months	NR	Reduced dose: 100% Standard dose: 96%	Reduced dose: 0% Standard dose: 0%	Reduced dose: 0% Standard dose: 4%	Reduced dose: 0% Standard dose: 0%	CTCAE	Ulceration, infection, odor ^d^	NR	NR
Bertino et al., 2016 [16]	RECIST	Lesion	2 months	6 months (15 days–12 months)	≤3 cm: 93.5% >3 cm: 66.7%	≤3 cm: 6.5% >3 cm: 0%	≤3 cm: 0% >3 cm: 33.3%	≤3 cm: 0% >3 cm: 0%	CTCAE	Ulceration, hyperpigmentation, suppuration, headache, odor, dysphagia, rash ^d^	1 year: 100%	1-year LDFS: 89%
Tomassini et al., 2016 [34] ^d^	RECIST	Lesion	2 months	NR	NS	NS	NS	NS	NR	NR	NR	NR
Claussen et al. 2022 [35] ^d^	RECIST	Lesion	1–2 months	Minimum of 180 days	NS	NS	NS	NS	CTCAE	Pain, hyperpigmentation	NR	NR
Lyons et al. 2023 [27]	RECIST	Lesion	12 weeks	18 months	85% ^h^	15% ^h^	NR	0% ^h^	NR	NR	NR	NR
Clover et al. 2020 [36]	RECIST	Lesion	At least 45 days	NR	85%	11%	NR	NR	NR	NR	NR	NR
Campana et al. 2017 [41]	RECIST	Patient	1 month	49.2 months (3.6–121.1)	50.0% ^i^	35.7% ^i^	14.3% ^i^	0% ^i^	CTCAE	Erythema, edema, pain, ulceration, infection	NR	5-year LPFS: 70%
Rotunno et al. 2018 [42]	RECIST	Lesion	60 days	165 days (60–1061)	83%	17%	0%	0%	NR	Pain, hyperpigmentation, ulceration, erythema, nausea, flu-like symptoms ^d^	NR	NS
Solari et al. 2014 [28] ^d^	RECIST	Patient	NR	NR	NS	NS	NS	NS	NR	NR	NR	NR

CR: complete response; CTCAE: Common Terminology Criteria for Adverse Events; ECT: electrochemotherapy; LDFS: local disease-free survival; LPFS: local progression-free survival; NR: not reported; NS: not specified; OS: overall survival; PD: progressive disease; PR: partial response; RECIST: Response Evaluation Criteria in Solid Tumors; SD: stable disease; ^a^ Response after one ECT session, CR was 83.3% after two sessions, 91.6% after four sessions, and 100% after five sessions; ^b^ Response after one ECT session, CR was 100% after two sessions; ^c^ Response after primary excision, CR was 100% after the 2nd further wider excision; ^d^ The data specific to BCC are not available and encompasses a broader category of skin cancers as reported in the individual studies; ^e^ Out of all evaluable lesions, three (1%) patients were unable to be evaluated due to the presence of inflammation or ulceration; ^f^ Out of all evaluable lesions, eight (1.4%) lesions were unable to be evaluated due to the presence of inflammation or ulceration; ^g^ Deaths were not related to disease; ^h^ Assessed 18 months after ECT; ^i^ Response after one ECT session, CR was 63% after two sessions.

**Table 6:** Tumor response and toxicity for squamous cell carcinoma (SCC).

Study	Response Scale	Response Evaluation	Time of Response Evaluation	Follow-Up Duration, Median (Range)	CR (%)	PR (%)	SD (%)	PD (%)	Toxicity Scale	Toxicity	OS	Local Tumor Control
Campana et al. 2016 [30]	RECIST	Patient	60 days	13.9 months (0.4–63.2)	40.7%	NR	NR	NR	CTCAE	Grade 0–1 (60%), 2–4 (40%)	NS	NS
Bertino et al. 2022 [37]	RECIST	Patient/Lesion	45–90 days	5.6 months (1.6–47.6)	62%/61%	21%/18%	11%/13%	5%/7%	CTCAE	Grade 1–2 (11%)	8.6 months: 85.1% ^b^	1-year LPFS: 80%/49% ^c^
Riva et al. 2021 [38] ^a^	RECIST	Patient	1 month	Up till 6 months	NS	NS	NS	NS	NR	Edema	NR	NR
Sersa et al. 2021 [39] ^a^	RECIST	Patient	38 and 80 days	≥90 years: 8 months (2–37) <90 years: 9 months (2–46)	NS	NS	NS	NS	CTCAE	Ulceration, odor, infection, hyperpigmentation	NS	NS
Jamsek et al. 2020 [32] ^a^	RECIST	Patient	2 months	Reduced dose: 28 months Standard dose: 40 months	Reduced dose: 100% Standard dose: 96%	NR	NR	NR	NR	NR	NR	NR
Bonadies et al. 2019 [40]	RECIST	Patient	2 months	NR	92%	8%	NR	NR	CTCAE	Necrosis, edema ^a^	NR	NS
Pichi et al. 2018 [26]	RECIST	Patient	1 month	7.6 months (2–18)	10%	90%	NR	NR	NR	Fever, pain ^a^	NS	NR
Groselj et al. 2018 [33]	RECIST	Lesion	2 months	NR	Reduced dose: 100% Standard dose: 100%	Reduced dose: 0% Standard dose: 0%	Reduced dose: 0% Standard dose: 0%	Reduced dose: 0% Standard dose: 0%	CTCAE	Ulceration, infection, odor ^a^	NR	NR
Bertino et al., 2016 [16]	RECIST	Lesion	2 months	6 months (15 days–12 months)	≤3 cm: 76.9% >3 cm: 28.6% ^d^	≤3 cm: 7.7% >3 cm: 42.9% ^d^	≤3 cm: 15.4% >3 cm: 14.3% ^d^	≤3 cm: 0% >3 cm: 9.5% ^d^	CTCAE	Ulceration, hyperpigmentation, suppuration, headache, odor, dysphagia, rash ^a^	1 year: 64%	1-year LDFS: 87%
Tomassini et al., 2016 [34] ^a^	RECIST	Lesion	2 months	NR	NS	NS	NS	NS	NR	NR	NR	NR
Kreuter et al. 2015 [43] ^a^	RECIST	Patient	NR	NR	NS	NS	NS	NS	NR	Pain, muscle ache, necrosis, hyperpigmentation, bleeding, infection	NR	NR
Claussen et al. 2022 [35]	RECIST	Lesion	1–2 months	Minimum of 180 days	<3 cm: 71% ^e^ >3 cm: 41.5% ^f^	<3 cm: 20.5% ^e^ >3 cm: 29.5% ^f^	<3 cm: 7.5% ^e^ >3 cm: 21.5% ^f^	<3 cm: 0.5% ^e^ >3 cm: 6.0% ^f^	CTCAE	Pain, hyperpigmentation ^a^	NR	NR
Lyons et al. 2023 [27]	RECIST	Lesion	12 weeks	18 months	100% ^g^	0% ^g^	NR	0% ^g^	NR	NR	NR	NR
Clover et al. 2020 [36]	RECIST	Lesion	At least 45 days	NR	63%	17%	NR	NR	NR	NR	NR	NR
Rotunno et al. 2018 [42]	RECIST	Lesion	60 days	165 days (60–1061)	86%	0%	14%	0%	NR	Pain, hyperpigmentation, ulceration, erythema, nausea, flu-like symptoms ^a^	NR	NS
Di Monta et al. 2017 [44]	RECIST	Patient	4 weeks	34 months (5–48)	22.7%	59.1%	13.6%	4.5%	CTCAE	Pain, erythema	NR	NR
Solari et al. 2014 [28] ^a^	RECIST	Patient	NR	NR	NS	NS	NS	NS	NR	NR	NR	NR

CR: complete response; CTCAE: Common Terminology Criteria for Adverse Events; ECT: electrochemotherapy; LDFS: local disease-free survival; LPFS: local progression-free survival; NR: not reported; NS: not specified; OS: overall survival; PD: progressive disease; PR: partial response; RECIST: Response Evaluation Criteria in Solid Tumors; SD: stable disease. ^a^ The data specific to SCC are not available and encompasses a broader category of skin cancers as reported in the individual studies. ^b^ In total, 16 (66.7%) deaths were not related to disease, 8 (33.3%) deaths were related to disease. ^c^ Patients with primary tumors and locally advanced disease. ^d^ One (4.7%) lesion was unable to be evaluated due to the presence of crust formation and ulceration. ^e^ 0.5% of lesions were not evaluated. ^f^ In total, 2% of lesions were not evaluated. ^g^ Assessed 18 months after ECT3.5.2. Overall Survival and Long-Term Local Tumor Control.

## Data Availability

Data of the current original research are available from the corresponding author on reasonable request.
